# Time Trends and Causes of Infection-Related Mortality Among Patients Starting Dialysis in Finland: A Nationwide Cohort Study

**DOI:** 10.1016/j.xkme.2025.101012

**Published:** 2025-04-18

**Authors:** Susanna Kinnunen, Ilkka Helanterä, Auni Juutilainen, Wisam Bitar, Jaakko Helve, Patrik Finne

**Affiliations:** 1 Division of Nephrology, Department of Medicine, Kuopio University Hospital and University of Eastern Finland, Kuopio, Finland; 2 Transplantation and Liver Surgery, Helsinki University Hospital and University of Helsinki, Helsinki, Finland; 3 Institute of Clinical Medicine/Internal Medicine, University of Eastern Finland, Kuopio, Finland; 4 Department of Nephrology, University of Helsinki and Helsinki University Hospital, Helsinki, Finland; 5 Finnish Registry for Kidney Diseases, Finnish Kidney and Liver Association, Helsinki, Finland

**Keywords:** Dialysis, hemodialysis, incidence rate, incidence rate ratio, infection-related mortality, kidney replacement therapy, mortality, peritoneal dialysis, sepsis

## Abstract

**Rationale & Objective:**

Previously, we reported a decrease in infection-related mortality in kidney transplant recipients. Regarding patients treated with dialysis, it is unclear whether infection-related mortality is decreasing. Therefore, we investigated current time trends and specific causes of infection-related mortality over 2 decades in a large cohort of patients treated with dialysis.

**Study Design:**

A nationwide cohort study.

**Setting & Participants:**

Patients starting kidney replacement therapy were identified through the Finnish Registry for Kidney Diseases. Follow-up continued until death of any cause, kidney transplantation, moving abroad, recovery of kidney function, loss of follow-up, or end of study.

**Exposure:**

Long-term kidney replacement therapy.

**Outcome:**

Death due to infection.

**Analytical Approach:**

Incidence rates, incidence rate ratios, and adjusted Cox regression hazard ratios for infection-related deaths were calculated by sub-cohorts consisting of patients whose kidney replacement therapy was started either 2000-2004, 2005-2009, 2010-2014, or 2015-2019. As sensitivity analyses, we studied infection-related mortality within 1 year of dialysis initiation and performed competing risk analysis.

**Results:**

A total of 9,671 adult patients started long-term dialysis from 2000 to 2019. Infection-related deaths declined from 47 to 23 deaths per 1,000 person-years over the four 5-year intervals from 2000-2004 to 2015-2019. The hazard ratio of a Cox model including identified risk factors was 0.49 (95% CI, 0.39-0.62) for patients who started dialysis in 2015-2019 compared with those who started in 2000-2004. The most common specific causes of infection-related deaths were septicemia (38%), pulmonary infection (36%), and peritonitis (8%), whereas opportunistic infections rarely caused death.

**Limitations:**

Death certificates may have low sensitivity for infectious diseases. Only one cause of death is available. Categories of infectious deaths may overlap.

**Conclusions:**

Risk of dying due to infections has halved since the beginning of the millennium despite aging among patients treated with dialysis. The reason for this development requires further studies.

## Plain Language Summary

Patients treated with dialysis experience a very significantly increased risk of dying from infection compared to the general population. This study evaluated current time trends and specific causes of infectious disease mortality in patients treated with dialysis in Finland from 2000 to 2019 based on data from the national registry with complete coverage of the dialysis cohort. The risk of death from infection in maintenance dialysis in Finland has dropped by half since the 2000s, despite the aging and increased number of patients treated with dialysis. Sepsis, pneumonia, peritonitis, and common bacterial infections were the most frequent causes of infectious deaths in patients treated with dialysis. This period of study preceded the Covid-19 pandemic and provides a baseline for future comparison.

Patients treated with dialysis are at a significantly higher mortality risk than the general population. Next to cardiovascular diseases, infections are the most common causes of death among patients treated with dialysis, accounting for about 10%-20% of all deaths.^1^^,^^2^

Studies based on data from the United States Renal Data System during 1990s showed that patients treated with dialysis had up to 100-fold age-adjusted mortality from sepsis and 10-fold risk of death from pulmonary infections in comparison to the general population.^3^^,^^4^ A more recent European study showed that patients treated with dialysis have an 82-fold risk of dying from infection compared with the general population.^5^ Of patients with a known causative agent, 96% died of bacterial infections, 2.6% from viral infections, and 1.4% from other types of infections. Detailed information about the specific types of infections was lacking in most previous studies, and the cause of death was largely unknown or missing.

We have earlier shown that the risk of death from infections after kidney transplantation has halved since the 1990s and that common bacterial infections remain the leading cause of infection-related death among transplant recipients.^6^ However, the literature on time trends and types of infectious diseases responsible for excess mortality in patients treated with dialysis provides widely varying results.^7^, ^8^, ^9^ Therefore, we used data from the Finnish Registry for Kidney Diseases to analyze the time trends in infection-related mortality and the causes of infectious deaths over a period of 2 decades.

## Methods

### Study Design

We conducted a retrospective epidemiological study of trends in infection mortality in patients treated with dialysis who started kidney replacement therapy (KRT) from 2000 to 2019. All data were retrieved from the Finnish Registry for Kidney Diseases, which covers 97%-99% of all patients accepted for KRT in Finland since 1965.^10^ All patients provided written informed consent and permission to use the data anonymously in registry reports and for research purposes. All Finnish dialysis units provide the registry with specific information on patients starting long-term KRT and annual follow-up data. This study adheres to the Declaration of Helsinki.

### Study Population

The study cohort included all adult (aged 18 years or older) incident patients who entered long-term dialysis treatment in Finland from January 1, 2000, to December 31, 2019. During the same period, 47 patients received a preemptive kidney transplantation but were not included in the study. We excluded patients whose kidney function had recovered within 90 days or who underwent kidney transplantation before dialysis.

### Data Collection

Data were collected on age, sex, cause of kidney failure, dialysis modality at start and changes in modality thereafter, date of first kidney transplantation, and time and cause of death. Data were also collected on comorbid conditions, such as coronary artery disease, peripheral vascular disease, heart failure, left ventricular hypertrophy, cerebrovascular disease, and body mass index classified according to WHO, and laboratory data. The cause of kidney failure was categorized into 4 groups: glomerulonephritis, polycystic kidney disease, diabetes type 1 or type 2, and other causes (tubulointerstitial nephritis, amyloidosis, nephrosclerosis, and other or unknown diagnoses).

Data were complete regarding outcome, explanatory variables (age, sex, cause of kidney failure, and dialysis modality) and cause of death, whereas data on comorbid conditions and laboratory findings were missing to varying degrees (1.0%-13.3%) (Item S1, Table S1).

### Follow-Up

Patients were followed from the start of dialysis until they died of any cause, received kidney transplantation, moved abroad, kidney function recovered, were lost to follow-up, or until December 31, 2019 (end of study), whichever occurred first.

### Causes of Death

The International Classification of Diseases (ICD)–10 was used for classifying the deaths. Causes of death were grouped into 4 categories (infection, cardiovascular, malignancy, and other), and infection-related deaths, further divided into 9 categories: septicemia, pulmonary infection defined as bacterial or unspecified pneumonia, pneumonitis or empyema/pleural effusion, peritonitis, cardiac, gastrointestinal, specified viral infection, specified fungal infection, tuberculosis, and other infections (Table S2).

Causes of death were reported to the Finnish Registry for Kidney Diseases by the treating nephrologist. The statistics on mortality in Finland is based on death certificates, where ICD‒10 code for cause of death is assigned by the physician who treated the deceased during the final illness. All death certificates shall be submitted for inspection by a forensic medicine expert of the competent authority. After approval, they are sent to the National Death Register at Statistics Finland. If the cause of death was not reported to the Finnish Registry for Kidney Diseases, it is obtainable from Statistics Finland based on personal social security numbers.

### Primary Outcome

The primary outcome was death due to infection, and its subcategories were death due to sepsis and early infectious death within 1 year of dialysis initiation.

### Statistical Methods

Descriptive analyses are presented as medians with interquartile ranges for continuous variables and counts with percentages for categorical variables. Comparisons between the groups were performed using the χ^2^ test for categorical variables and the Mann-Whitney U test or the Kruskal-Wallis for continuous variables. Incidence rates and incidence rate ratios for infection-related deaths with 95% confidence intervals (CI) were calculated over the four 5-year periods from 2000-2004 to 2015-2019 by sub-cohorts based on dialysis initiation year. We used Cox proportional hazards regression model with multivariable adjustment to estimate hazard ratios (HRs) for death from infection. Variables significant in the univariable model (*P* < 0.05) were selected to the multivariable model. In these analyses, patients were censored at time of transplantation, death due to other cause, end of follow-up, or at the latest at 5 years from start of dialysis. To enable the comparison of different eras, a limit of 5 years was used. Trends over time were tested either with unweighted linear regression^11^ or the χ^2^ test for trend. IBM SPSS Statistics (version 27.0.0) was used for statistical analyses, and the software R version 4.2.3 to calculate cumulative incidence by Fine-Gray subdistribution hazard regression. Two-sided *P* values lower than 0.05 were considered statistically significant.

### Sensitivity Analysis

The sensitivity analysis outcome was early infectious death within 1 year of dialysis initiation. As another sensitivity analysis, we used Fine-Gray subdistribution hazard model for infection-related death to account for kidney transplantation and other causes of death as competing risk events.

## Results

During the period 2000-2019, 9,671 adult patients (aged ≥18 years) started KRT and were included in the study. Patients were followed for 5 years from the start of KRT until they died of any cause (38%, n = 3,692), received a kidney transplant (30%, n = 2,909), were lost to follow-up (0%, n = 1), moved abroad (0.1%, n = 11), kidney function recovered (1.8%, n = 176), or until the end of the 5 year follow-up period on December 31, 2019 (end of study, 30%, n = 2,882). The median follow-up time was 2.3 years for the 2 earlier and 1.9 years for the 2 recent cohorts. Overall patient survival at 1 year in the entire dialysis cohort was 90% during the 2 recent and 88% during the 2 earlier cohorts, and 44% and 38% at 5 years, respectively.

Compared with earlier 5-year periods, patients recruited during more recent periods were older, more often male, and had a higher body mass index but less comorbid conditions, such as ischemic heart disease and peripheral vascular disease (Table 1). Kidney transplantation became more frequent (Table 1). Levels of albumin, hemoglobin, and C-reactive protein decreased over the 5-year periods from 2000 to 2019. In addition, hemodiafiltration increased markedly over the 5-year periods from 2000 to 2019 (0.2%-0.9%-3.6%-9.8%). In fully adjusted analyses, earlier era of KRT initiation, older age, low plasma albumin, peripheral vascular disease, and chronic heart failure were independently associated with both death from infection (Table S3) and death from sepsis (Table S4). As to patients treated with hemodialysis (HD) and peritoneal dialysis (PD), the risk of death from infection was similar in the less adjusted Cox model but higher in patients treated with PD in the fully adjusted model.

**Table 1:** Patient Characteristics for the 4 Study Periods from 2000 to 2019

Characteristics	All N = 9,671	2000-2004 n = 2,398	2005-2009 n = 2,376	2010-2014 n = 2,273	2015-2019 n = 2,624	*P*
Male sex	6,301 (65)	1,488 (62)	1,555 (65)	1,552 (68)	1,706 (65)	0.009
Age at dialysis onset	64 (52-72)	62 (50-71)	63 (52-73)	64 (53-73)	65 (53-73)	< 0.001
Age group at dialysis onset						< 0.001
18-44 y	1,375 (14)	385 (16)	346 (15)	289 (13)	355 (13)	
45-64 y	3,842 (40)	1,001 (42)	964 (40)	915 (40)	962 (37)	
65-74 y	2,654 (27)	647 (27)	593 (25)	628 (28)	786 (30)	
75+ y	1,800 (19)	365 (15)	473 (20)	441 (19)	521 (20)	
Cause of KRT						0.12
Glomerulonephritis	1,337 (14)	326 (14)	340 (14)	290 (13)	381 (15)	
Polycystic kidney	943 (10)	219 (9)	203 (9)	235 (10)	286 (11)	
Diabetes	3,351 (34)	849 (35)	832 (35)	794 (35)	876 (33)	
Other	4,040 (42)	1,004 (42)	1,001 (42)	954 (42)	1,081 (41)	
Initial treatment modality						0.04
Hemodialysis	7,288 (75)	1,851 (77)	1,754 (74)	1,726 (76)	1,957 (75)	
Peritoneal dialysis	2,383 (25)	547 (23)	622 (26)	547 (24)	667 (25)	
Treatment modality at 90 days from start of dialysis						< 0.001
Hemodialysis	6,796 (72)	1,680 (73)	1,647 (71)	1,590 (72)	1,879 (73)	
In-center HD	6,145 (65)	1,618 (71)	1,576 (68)	1,435 (65)	1,516 (59)	
HDF	357 (3.8)	5 (0.2)	20 (0.9)	80 (3.6)	252 (9.8)	
Home HD	294 (3.1)	57 (2.5)	51 (2.2)	75 (3.4)	111 (4.3)	
Peritoneal dialysis	2,549 (27)	595 (26)	662 (28)	621 (28)	671 (26)	
APD	1,256 (13)	202 (9)	362 (16)	331 (15)	361 (14)	
CAPD	1,293 (14)	393 (17)	300 (13)	290 (13)	310 (12)	
Transplantation	69 (0.7)	20 (0.9)	13 (0.6)	7 (0.3)	29 (1.1)	
Transplantation within 5 years from start of dialysis	2,909 (30)	795 (33)	716 (30)	792 (35)	606 (23)	< 0.001
Time to kidney transplant from start of dialysis (d)	580 (355-945)	541 (312-917)	618 (393-1014)	646 (420-1024)	500 (301-842)	< 0.001
Comorbid condition						
Ischemic heart disease	2,423 (25)	641 (27)	638 (27)	568 (25)	576 (22)	< 0.001
Chronic heart failure	1,065 (11)	277 (12)	257 (12)	237 (10)	275 (11)	0.37
Peripheral vascular disease	1,452 (15.0)	371 (16)	355 (15)	378 (17)	348 (13)	0.010
Stroke	1,096 (11)	248 (10)	301 (13)	255 (11)	292 (11)	0.08
Weight status BMI kg/m^2^						<0.001
<25 normal	3,795 (39)	1,064 (44)	980 (41)	847 (37)	904 (34)	
25-29.9 overweight	3,292 (34)	852 (36)	788 (33)	740 (33)	912 (35)	
≥30 obese	2,584 (27)	482 (20)	608 (26)	686 (30)	808 (31)	
Laboratory findings^a^						
P-Albumin (g/dL)	3.3 (2.8-3.7)	3.3 (2.8-3.7)	3.3 (2.8-3.7)	3.2 (2.7-3.6)	3.2. (2.7-3.6)	< 0.001
P-Phosphate (mg/dL)	5.6 (4.6-6.8)	5.6 (4.6-6.8)	5.6 (4.5-8.8)	5.7 (4.6-6.9)	5.5 (4.6-6.7)	0.014
P-Creatinine (mg/dL)	6.5 (5.2-8.2)	6.5 (4.9-8.2)	6.4 (7.1-8.3)	6.6 (5.3-8.3)	6.6 (5.4-8.1)	0.025
B-Hemoglobin (g/dL)	10.5 (9.5-11.5)	10.7 (9.6-11.8)	10.8 (9.8-11.9)	10.4 (9.4-11.4)	10.3 (9.4-11.2)	< 0.001
C-reactive protein (mg/L)	8.0 (3.0-30.0)	11.7 (5.0-40.0)	8.0 (3.0-27.0)	7.1 (3.0-27.9)	6.0 (2.0-24.7)	< 0.001

*Note:* Number of patients (%) or median (interquartile range).

Abbreviations: APD, automated peritoneal dialysis; BMI, body mass index; CAPD, continuous ambulatory peritoneal dialysis; HD, hemodialysis; HDF, hemodiafiltration; KRT, kidney replacement therapy.

a At the start of kidney replacement therapy.

### Trends in Infection-Related Mortality From 2000-2004 to 2015-2019

**Figure 1:**
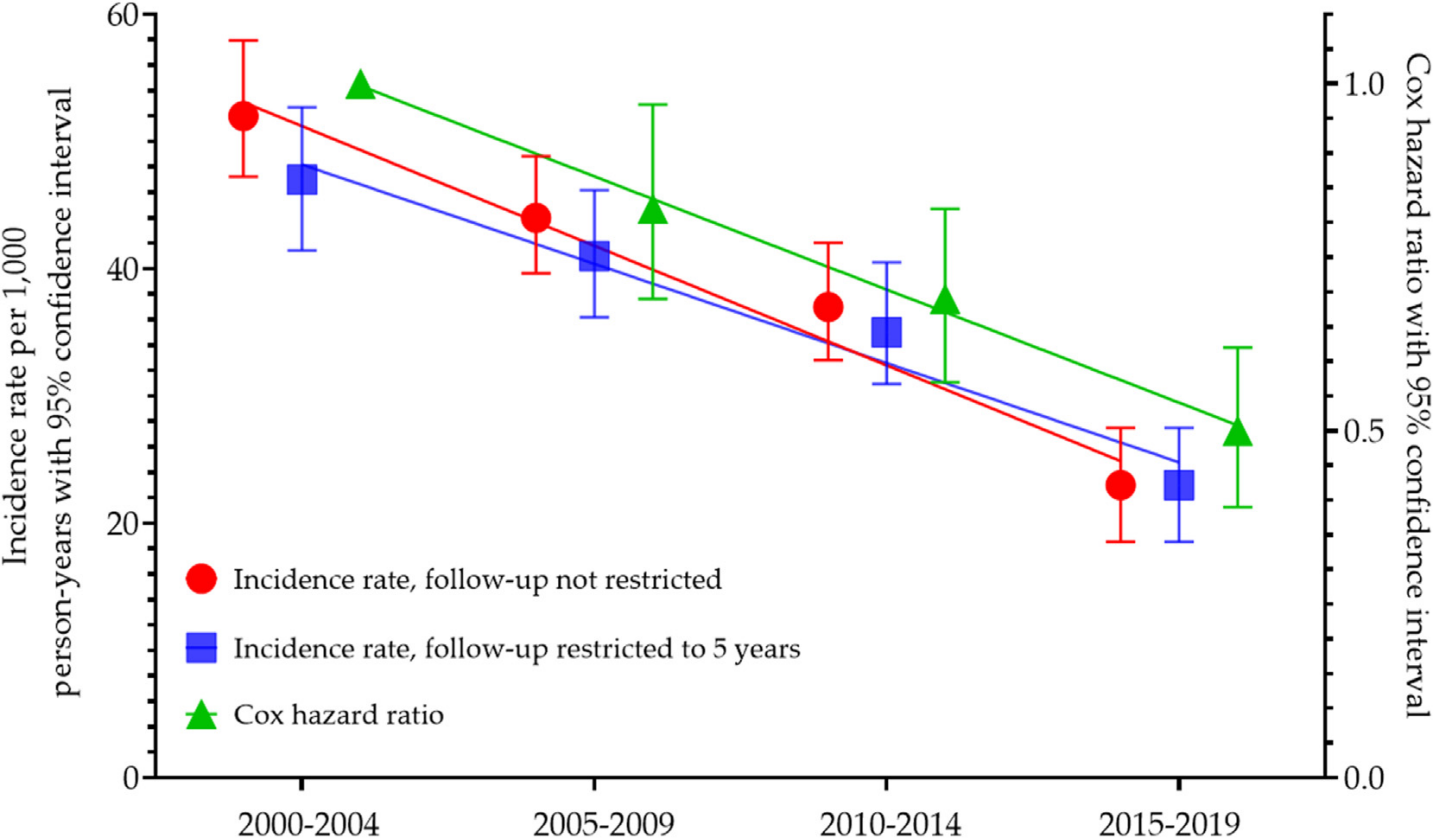
Time trend of infection-related mortality in patients on kidney replacement therapy, depicted by incidence rates and Cox hazard ratios.

**Figure 2:**
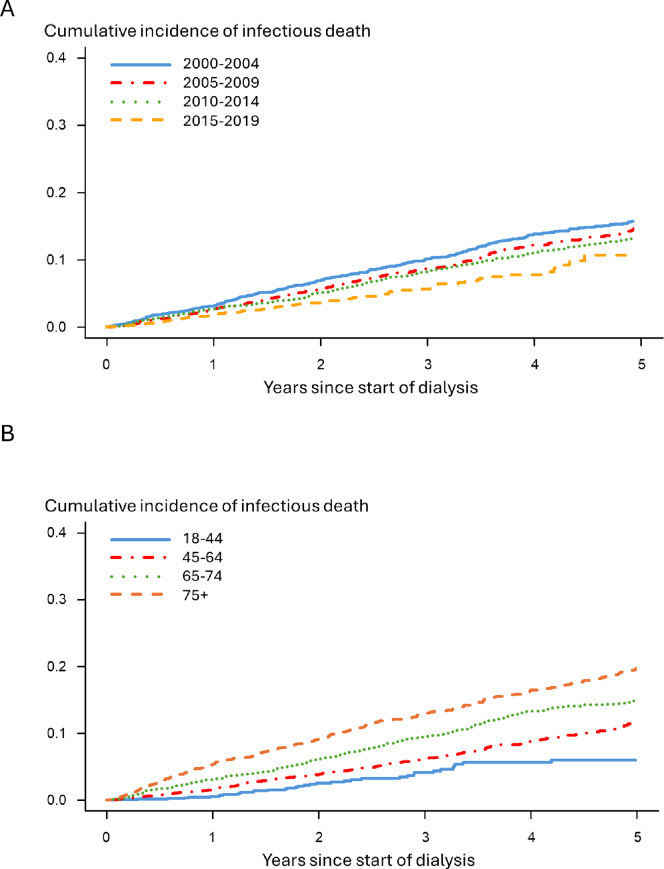
(A) Cumulative incidence of death due to infection by start year of dialysis; and (B) cumulative incidence of infection-related death in dialysis population by age groups (years) at start of dialysis.

To clarify the trend in major cause of death categories (infection, cardiovascular, malignancy, and other), we provide Table S5, and in infectious death categories, Table S6. The incidence rates and incidence rate ratios of infection-related deaths decreased steadily over the five-year intervals from 2000-2004 to 2015-2019 (incidence rates from 52-44-37-23 deaths per 1000 person-years and incidence rate ratios from 1 to 0.84-0.71-0.43, respectively) (Fig 1; Table 2 and Table S7). Cumulative incidence of death due to infections decreased consistently over time periods (Fig 2A). The rates of cumulative incidence of deaths were lower in younger age groups (Fig 2B), The unadjusted HR of infectious deaths was 0.54 (95% CI, 0.43-0.68) for patients who started maintenance dialysis in 2015-2019 compared with those who started in 2000-2004 (Table 2). After adjustment for age and sex, the risk of infectious mortality was 51% lower in the 2015-2019 cohort than in 2000-2004 cohort (HR 0.49; 95% CI, 0.39-0.62). Further multivariable adjustment did not change the results (HR 0.49; 95% CI, 0.39-0.62) (Table S3). Correspondingly, the risk of death from sepsis decreased by 50% (HR 0.50, 95% CI, 0.35-0.71) from 2000-2005 to 2015-2019 (Table S4).

**Table 2:** Time Trends in Infection-Related Mortality by 5-Year Periods of Kidney Replacement Treatment Initiation From 2000 to 2019

	2000-2004	2005-2009	*P*_1_	2010-2014	*P*_1_	2015-2019	*P*_1_	*P*_2_
Cox model hazard ratios								
Unadjusted	1	0.86 (0.73-1.02)	0.076	0.75 (0.63-0.89)	0.001	0.54 (0.43-0.68)	< 0.001	
Adjusted for age and sex	1	0.81 (0.69-0.96)	0.016	0.68 (0.57-0.82)	0.001	0.49 (0.39-0.62)	< 0.001	
Multivariable adjustment^a^	1	0.81 (0.68-0.95)	0.012	0.67 (0.56-0.80)	< 0.001	0.49 (0.39-0.62)	< 0.001	0.002
Event rates per person-years without restricting follow-up to 5 years								
Infection-related deaths	377	363		260		104		
Patient-years	7,194.78	8,235.166		6,983.282		4,582.181		
IR per 1,000 person-years	52	44		37		23		0.013
IRR (95% CI)	1 (ref.)	0.84 (0.76-0.93)		0.71 (0.64-0.79)		0.43 (0.39-0.48)		
Event rates per person-years restricting follow-up to 5 years								
Infection-related deaths	275	267		220		104		
Patient-years	5,871.69	6,516.92		6,198.12		4,582.2		
IR per 1,000 person-years	47	41		35		23		0.017
IRR (95% CI)	1 (ref.)	0.86 (0.74-1.04)		0.76 (0.64-0.91)		0.49 (0.39-0.61)		

IR, incidence rate; IRR, incidence rate ratio; *P*_1_, in comparison to 2000-2004; *P*_2_*,* significance is tested by linear regression adopted by Brownstein and Cai (2019).^11^

a Adjusted for age at dialysis initiation, sex, cause of kidney disease, weight status (BMI), peripheral vascular disease, coronary artery disease, chronic heart failure, cerebrovascular disease, serum albumin.

### Causes of Infection-Related Mortality

Infections accounted for 23% of all deaths and their proportion decreased from 23.8% (275/1156) in 2000-2004 to 19.5% (104/532) in 2015-2019 (Table S5). During the 5 years of follow-up, septicemia and pulmonary infection were the most frequent causes of infection-related mortality, with decreasing trends over the four 5-year periods from 2000 to 2019 (Table 3). The proportion of deaths due to pulmonary infections among all infectious deaths decreased slightly (39%-37%-32%-31%; *P* = 0.051), whereas the proportion of sepsis deaths showed no clear trend (39%-35%-41%-40%) over the 5-year periods from 2000 to 2019. Septicemia caused 333 deaths, of which the causative pathogen was known in 209 (63%) of the cases, while 124 causes of deaths (37%) were unspecified septicemias. *Staphylococcus aureus* accounted for 93 (44%), other staphylococci for 22 (11%), gram-negative sepsis for 36 (17%) and *streptococcal* septicemia for 12 (5.7%) of the cases with known microbiological etiology (Table S2).

**Table 3:** Trends by Subgroups of Infection-Related Deaths Among Patients treated with Dialysis Whose Dialysis was Started in 2000-2004, 2005-2009, 2010-2014, or 2015-2019

Cause of Infectious Death	All N = 9,671	2000-2004 n = 2,398	2005-2009 n = 2,376	2010-2014 n = 2,273	2015-2019 n = 2,624	*P*_1_	*P*_2_
Septicemia	333 (39)	106 (39)	94 (35)	91 (41)	42 (40)	0.48	< 0.001
Pulmonary infection	310 (36)	108 (39)	99 (37)	71 (32)	32 (31)	0.051	< 0.001
Peritonitis	73 (8)	24 (9)	26 (10)	15 (7)	8 (8)	0.48	0.001
Cardiac infection	27 (3)	6 (2)	8 (3)	10 (5)	3 (3)	0.31	0.46
Gastroenteric infection	29 (3)	8 (3)	9 (3)	9 (4)	3 (3)	0.73	0.17
Fungal infection	11 (1)	0 (0)	7 (3)	4 (2)	0 (0)	0.54	0.63
Viral infection	7 (1)	1 (0.4)	0 (0)	3 (1)	3 (3)	0.011	0.15
Tuberculosis	4 (0.5)	1 (0.4)	0 (0)	1 (0.5)	2 (2)	0.10	0.41
Other infection	72 (8)	21 (8)	24 (9)	16 (7)	11 (11)	0.60	0.028
All infections	866	275	267	220	104	-	<0.001

*P*_1_, χ^2^ trend test for time-dependent change in the fraction of total infectious deaths; *P*_2_, χ^2^ trend test for time-dependent change in the fraction of total number of patients. N (% of all infectious deaths).

Septicemias and pulmonary infections were more frequent in patients starting HD and peritonitis in patients starting PD (Table 4). Peritonitis was the cause of death in 73 patients (8.4% of all infectious deaths). Cardiac infection (pericarditis, endocarditis, and myocarditis) caused 3.1% and gastroenterological infection 3.3% of the infectious deaths. Enterocolitis due to *Clostridium difficile* accounted for 38% of the gastroenterological deaths. Of the 72 deaths categorized as other infections, 60% (n = 43) were caused by other specified or unspecified bacterial infections (Table S2). Invasive fungal, viral, or opportunistic bacterial infections rarely caused death. Fungal infections comprised 1.3% of all infectious deaths. There were few fatal viral infections, 5 deaths due to influenza virus and 1 fatal case of cytomegalovirus pneumonitis. *Listeria monocytogenes* caused 3 deaths and *Mycobacterium tuberculosis* 4 deaths. Isolated cases of *Varicella zoster* virus encephalitis, *Varicella zoster* virus pneumonia, and pneumonia due to *Pseudomonas* occurred as the cause of death after 5 years of follow-up.

**Table 4:** The First Dialysis Modality and Infection-Related Deaths

Dialysis Modality	All n = 866	Hemodialysis n = 688	Peritoneal Dialysis n = 178	*P* value for difference^a^
Septicemia	333 (38.5)	280 (40.7)	53 (29.8)	0.007
Pulmonary infection	310 (35.8)	259 (37.6)	51 (28.7)	0.028
Peritonitis	73 (8.4)	27 (3.9)	46 (25.8)	< 0.001
Cardiac infection	27 (3.1)	24 (3.5)	3 (1.7)	0.33
Gastroenteric infection	29 (3.3)	25 (3.6)	4 (2.2)	0.049
Fungal infection	11 (1.3)	8 (1.2)	3 (1.7)	0.71
Viral infection	7 (0.8)	6 (0.9)	1 (0.6)	1.00
Tuberculosis	4 (0.5)	3 (0.4)	1 (0.6)	1.00
Other infection	72 (8.3)	57 (8.1)	16 (9.0)	0.76

*Note:* n (% of all infectious deaths).

a Fisher’s exact test.

### Sensitivity Analyses

While restricting the follow-up to 1 year, 1,100 deaths occurred, and of these, infections accounted for 21% (n = 232). Sepsis (45%, n = 104) and pulmonary infections (34%, n = 79) were the most common causes of infectious death (Table S8). The 1-year adjusted risk of early infectious death after KRT initiation decreased by 53% between the earliest and the most recent cohort (HR 0.47; 95% CI, 0.32-0.69) (Table S8). When using the Fine-Gray method to account for kidney transplantation and death due to other causes as competing risks, the results on infection-related mortality remained concordant (Table S9) with the Cox regression models shown in Table 2. We also performed an analysis without restricting the follow-up time to 5 years, and the result remained practically unaltered (Table 2). Based on sensitivity analyses, including the Fine-Gray competing risk model, the result is robust.

## Discussion

In this nationwide cohort study involving 9,671 patients who started dialysis in Finland from 2000 to 2019, we found a prominent decrease in the risk of infectious death in dialysis population. The risk of death decreased by 50% in 2 decades, despite the increase in the age at the start of dialysis. Bacterial septicemia, pulmonary infections, and peritonitis accounted for most infectious deaths, whereas invasive fungal, viral, or opportunistic infections rarely caused death.

Infection is the leading cause of noncardiovascular mortality among patients treated with dialysis accounting for 10%-20% of deaths in Europe, the United States, and Japan.^1^^,^^5^^,^^12^ Compared with the general population, infection-related mortality is up to 100 times higher in patients treated with dialysis.^4^^,^^5^ A large European study of patients with chronic kidney disease published in 2009 showed that the age-adjusted risk of cardiovascular and noncardiovascular mortality was increased to a similar extent.^1^ Previous studies have demonstrated that the decline in mortality from KRT-dependent kidney failure is predominantly due to a reduction in cardiovascular deaths.^7^^,^^13^ Data from the European Renal Association Registry in patients who started KRT from 2002-2015 showed a 16% reduction in excess all-cause mortality relative to the general population over 5 years, and this was mainly the result of a decrease in atheromatous cardiovascular disease as the cause of death (28%), whereas infection-related mortality decreased to a lesser extent (10%).^8^ A large Danish study reported temporal changes from 1996 to 2017 in infectious and cardiovascular morbidity and mortality over 2 decades. Its findings were the opposite of ours: an increasing trend in infections was observed, especially in pneumonia and sepsis, although cardiovascular disease decreased in patients treated with HD and PD.^14^

The current understanding of time trends in infection-related mortality and the specific causes of infectious deaths is partly contradictory. Several earlier studies have not found a reduction in infection-related mortality. A marked decrease was observed in the proportion of cardiovascular deaths based on the United Kingdom Renal Registry, but infection as a cause of death remained constant accounting for 17%-20% of all deaths among patients treated with dialysis during 2000-2019.^15^ In a recent study from Australia and New Zealand, which included all adult patients who started dialysis between 1980 and 2018, the cause of death was infection in 12%, representing a marked decline in the excess mortality compared with the general population from around 100-fold in the 1980s to present around 20-fold.^16^ Interestingly, a large study of mortality trends among Japanese dialysis patients between 1988 and 2013 found improved survival, mainly due to a decrease in cardiovascular mortality, while the proportion of deaths from infectious diseases had remained constant at 17.5% for 25 years.^9^ In our population treated with dialysis, we observed a decrease in the share of deaths from infection among all deaths from 24% in 2000-2004 to 19% in 2015-2019 and a marked reduction in the incidence rates and incidence rate ratios of infectious deaths over the 5-year eras from 2000 to 2019.

A study from the European Renal Association Registry showed that cardiovascular mortality in dialysis patients decreased by 25%, while infection-related mortality decreased by only 8% between 1998–2002 and 2003–2007.^7^ However, a sensitivity analysis of renal registry data, in which less than 20% of deaths were missing or unknown, showed no statistically significant change in the risk of death from infection in people of age <65 years.^7^ These differences in findings may be partly explained by variations in registry coding practices and differences in the proportion of unknown or missing data. No cause of death was available for 32% of patients treated with dialysis who died in the United Kingdom^15^ and for nearly a quarter in 2019 in the United States.^17^ Notably, we had complete coverage of causes of death and were able to show that the risk of infectious deaths has decreased over the past 2 decades. Moreover, we observed a similar risk reduction in 1-year mortality and in longer follow-up.

In the United States in 2019, infection was the cause of death in 9.1% of patients treated with HD and 12.3% of patients treated with PD whose cause of death was known.^17^ A large study from Australia and New Zealand found that the cause of death was infection in 185 (95% CI, 178-191)/10,000 person-years in patients treated with HD and 232 (95% CI, 221-244)/10,000 person-years in patients treated with PD.^16^ This finding is consistent with our result suggesting higher infection-related mortality in patients treated with PD. According to a recent Cochrane Database review comparing the effectiveness of PD and HD on cause-specific mortality and incidence of bacteremia, any differences remained uncertain.^18^ We recognize the difficulties associated with the evaluation of the impact of treatment modality on infection-related mortality, eg, because the impact of treatment modality changes can be difficult to take into account in research.

A US Renal Data System-based cohort study of KRT patients showed that during 7 years of follow-up, 11.7% of all patients treated with HD and 9.4% of patients treated with PD had at least 1 septicemia.^19^ In a more recent retrospective study (N = 322,734), Sakhuja et al identified patients treated with maintenance dialysis with severe sepsis from a nationwide hospital database in the United States between 2005 and 2010.^20^ Compared with 2005, the adjusted odds ratio for mortality among patients treated with dialysis admitted for treatment of severe sepsis decreased by 46% by 2010, which is consistent with our results. According to the United States Renal Data System 2021 Report, among patients who died in 2019, the cause of death was septicemia in 6.5% of patients treated with HD and 9.4% of patients treated with PD.^21^

As in earlier studies from Finland, *Staphylococcus aureus* was the most prevalent pathogen of infectious deaths among patients treated with dialysis. Within the deaths attributed to a known causative microbial agent, gram-positive bacteria were found in 62.4% and gram-negative in 19.8% of the cases, which is in line with previous studies in patients treated with HD.^22^, ^23^, ^24^, ^25^ *Staphylococcus aureus* and, less commonly, *Staphylococcus epidermidis* are the predominant pathogens in access-related infections.^25^^,^^26^

The high rate of infections and infectious deaths can be attributed to multiple conditions surrounding KRT, such as older age, diabetes, and cardiovascular disease.^27^ The underlying mechanisms point to various aspects of immune system impairment in chronic kidney disease, such as impaired leukocyte function, endothelial dysfunction, accumulation of inflammatory cytokines, and increased oxidative stress.^28^, ^29^, ^30^, ^31^, ^32^, ^33^ The risk of infection is further exacerbated by the dependence of vascular access and PD catheters for maintenance dialysis.^34^ Vascular access is one of the most important quality indicators of HD. The proportion of patients treated with HD with fistula or graft decreased, from 87% in 2009 to 83% in 2019 in Finland among patients who had received KRT for at least 1 year.^35^

Although the reasons for the decreased risk of death from infection in the population treated with dialysis cannot unequivocally be determined from our data, there are several possible explanations. One of them could be more conservative treatment choice when deciding on dialysis of frail patients, as frailty significantly increases mortality in chronic kidney disease, especially in patients treated with dialysis.^36^^,^^37^ Improvements in infection prevention and treatment, such as timely diagnosis and administration of antibiotics, may have contributed to the decline in infection-related mortality of patients treated with dialysis. Miskulin et al^38^ found that change in comorbid condition level over time was significantly associated with mortality in patients treated with dialysis. Therefore, better management and treatment of comorbid conditions before dialysis initiation may have contributed to improved survival. Despite the increase in the age of dialysis initiation, the percentage of patients with ischemic heart disease had decreased suggesting better cardiovascular health in people entering KRT.

The proportion of patients on in-center HD in Finland has remained stable, but the proportion of patients on hemodiafiltration has increased. The results from the CONVINCE trial support the evidence that high-dose hemodiafiltration can result in a clinically important survival benefit and reduction in all-cause mortality, mostly driven by a decrease in infection-related mortality (HR 0.69; 95% CI, 0.49-0.96).^39^

Even though sepsis incidence and sepsis mortality have increased in the general population in Finland and in other industrialized countries,^40^^,^^41^ we observed a decline in infection-related and sepsis mortality among patients treated with dialysis and the result was confirmed by sensitivity analyses. A major strength of this study is the long follow-up period of 20 years and the large nationwide study population with nearly 100% coverage of patients entering KRT during the study period. Data were collected and coded in an identical manner throughout the study period and mortality data were complete. Therefore, the possibility of selection or information bias is minimal. Patients who received a preemptive transplant were not included, but because this procedure was rare, our cohort stands for the entire incident KRT cohort, including patients who subsequently received a transplant. This adds to the generalizability of the study results.

A few limitations should be noted. Death certificates, although a widely used and accepted tool in population-based research because of their uniform availability, may have limitations, such as low sensitivity for infectious diseases. Although patients may have several factors contributing to death, only one cause of death is reported to the Finnish Registry for Kidney Diseases. Furthermore, there may be overlap between infection-related death categories. However, earlier studies have verified the validity and quality of death certificates produced by the register.^42^ Our multivariable models included only baseline variables at the start of dialysis. Explicit data on changes in frailty over time were lacking. The progression of comorbid conditions and social determinants of health influencing the risk of severe infection were not considered. Unfortunately, vaccination data for the study cohort were not available, although vaccination rates may have influenced infectious disease mortality. Our nationwide analysis is restricted to one geographical region with predominantly White population and one health care system, so the results may not be generalizable to other countries.

To conclude, infection-related mortality has decreased in patients on maintenance dialysis over 2 decades. Remarkably, this has occurred despite an aging population treated with dialysis and increase in the number of patients starting dialysis, possibly reflecting greater acceptance of patients onto dialysis treatment. Understanding the reason for this development requires further studies.

## Supplementary Materials

Supplementary File (PDF) Item S1; Tables S1-S9.

Supplementary Materials Table of Contents
