# Supplementary material for: Time Trends and Causes of Infection-Related Mortality Among Patients Starting Dialysis in Finland: A Nationwide Cohort Study
Source: Kidney Med. 2025 Apr 18;7(6):101012. doi: 10.1016/j.xkme.2025.101012 (PMC12152325; doi:10.1016/j.xkme.2025.101012)
Supplement: Supplementary Materials — Table of Contents [file mmc2.pdf]

**Supplementary Materials**

Table of Contents

**Item S1:** Imputation of Missing Values.

**Table S1:** Missing Values (%) at the Start of Kidney Replacement Therapy.

**Table S2:** Causes of Infection-Related Deaths Classified According to ICD-10 in Patients Treated With Maintenance Dialysis.

**Table S3:** Adjusted Cox Regression Analysis for Infection-Related Mortality in Patients Treated With Maintenance Dialysis.

**Table S4:** Adjusted Cox Regression Analysis for Sepsis Mortality in Patients Treated With Maintenance Dialysis.

**Table S5:** Cause of Death by Main Categories for the 4 Study Periods From 2000 to 2019 as Fraction of all Patients Treated With Maintenance Dialysis.

**Table S6:** Causes of Infectious Deaths over Four 5-Year Study Periods From 2000 to 2019 as Fraction of all Patients Treated With Maintenance Dialysis.

**Table S7:** Linear Regression Analysis of Time Trends for Infection-Related Mortality Based on Cox Hazard Ratios and Incidence Rates with 5-Year Restricted and Non-Restricted Follow-up From 2000 to 2019.

**Table S8:** Adjusted Cox Regression Analysis for Early Infection-Related Mortality Within one Year From Dialysis Initiation.

**Table S9:** Time Trends of Infection-Related Risk of Death by Fine-Gray Model Taking into Account Kidney Transplantation and Death From Other Causes as Competing Risks.

**Item S1.** Imputation of Missing Values

To reduce selection bias, missing values were imputed statistically using multiple imputation based on the following predictors: sex, age at the start of kidney replacement therapy, cause of kidney failure, plasma creatinine, albumin, phosphate, C-reactive protein, hemoglobin, body mass index, angina pectoris, myocardial infarction in the past, history of coronary by-pass surgery or angioplasty, left ventricular hypertrophy, chronic heart failure, symptomatic peripheral vascular disease, surgery because of peripheral vascular disease, amputation because of peripheral vascular disease, cerebral infarction or hemorrhage, and infectious death.

**Table S1**. Missing Values (%) at the Start of Kidney Replacement Therapy

| **Characteristic** | **All**<br>**N=9,671** | **Missing values (%)** |
|---|---|---|
| Male gender | 6301 (65) | 0.0 |
| Age at dialysis onset | 64 (52–72) | 0.0 |
| Cause of kidney replacement therapy | | 0.0 |
| Initial treatment modality | | 0.0 |
| Treatment modality at 90 days from start of dialysis | | 0.0 |
| Comorbid condition | | |
| Ischemic heart disease | 2423 (25) | 4.4 |
| Chronic heart failure | 1065 (11) | 6.0 |
| Peripheral vascular disease | 1452 (15.0) | 4.9 |
| Stroke | 1096 (11) | 4.1 |
| Weight status, body mass index (kg/m$^2$) | | 5.3 |
| Laboratory findings [a] | | |
| P-Albumin (g/dL) | 3.3 (2.8–3.7) | 4.8 |
| P-Phosphate (mg/dL) | 5.6 (4.6–6.8) | 2.8 |
| P-Creatinine (mg/dL) | 6.5 (5.2–8.2) | 1.0 |
| B-Hemoglobin (g/dL) | 10.5 (9.5–11.5) | 2.0 |
| C-reactive protein (mg/L) | 8.0 (3.0–30.0) | 13.3 |

**Table S2.** Causes of Infection-Related Deaths Classified According to ICD-10 in Patients Treated With Maintenance Dialysis

| Type of infection | Cause of death (ICD–10) | | All n = 866 | 2000–2009 n=542 | 2010–2019 n=324 |
|---|---|---|---|---|---|
| Septicemia (N=333) | Septicemia, all | | 333 | 200 | 133 |
| | A40.0 | Septicemia due to Streptococcus, group A | 2 | 1 | 1 |
| | A40.1 | Septicemia due to Streptococcus, group B | 1 | | 1 |
| | A40.3 | Septicemia due to *Streptococcus pneumoniae* | 3 | 1 | 2 |
| | A40.8 | Other streptococcal septicemia | 5 | 3 | 2 |
| | A40.9 | Streptococcal septicemia, unspecified | 1 | 0 | 1 |
| | A41 | Other septicemia | 19 | 11 | 8 |
| | A41.0 | Septicemia due to *Staphylococcus aureus* | 93 | 49 | 44 |
| | A41.1 | Septicemia due to other specified staphylococcus | 20 | 13 | 7 |
| | A41.2 | Septicemia due to unspecified staphylococcus | 2 | 2 | 0 |
| | A41.4 | Septicemia due to anaerobes | 2 | 2 | 0 |
| | A41.5 | Septicemia due to other Gram-negative organisms | 36 | 13 | 23 |
| | A41.8 | Other specified septicemia | 25 | 16 | 9 |
| | A41.9 | Septicemia, unspecified | 124 | 89 | 35 |
| Pulmonary infection (n= 310) | Pulmonary infection, all | | 310 | 207 | 103 |
| | J13 | Pneumonia due to *Streptococcus pneumoniae* | 3 | 2 | 1 |
| | J15 | Bacterial pneumonia, not elsewhere classified | 2 | 2 | 0 |
| | J15.8 | Other bacterial pneumonia | 3 | 3 | 0 |
| | J15.9 | Bacterial pneumonia, unspecified | 53 | 43 | 10 |
| | J16.8 | Pneumonia due to other specified infectious organisms | 1 | 1 | 0 |
| | J18 | Pneumonia, organism unspecified | 7 | 6 | 1 |
| | J18.0 | Bronchopneumonia, unspecified | 15 | 13 | 2 |
| | J18.1 | Lobar pneumonia, unspecified | 3 | 3 | 0 |
| | J18.8 | Other pneumonia, organism unspecified | 2 | 0 | 2 |
| | J18.9 | Pneumonia, unspecified | 197 | 119 | 78 |
| | J20.9 | Acute bronchitis, unspecified | 2 | 2 | 0 |
| | J22 | Unspecified acute lower respiratory infection | 1 | 1 | 0 |
| | J44.0 | Chronic obstructive pulmonary disease with acute lower respiratory infection | 2 | 0 | 2 |
| | J69 | Pneumonitis due to solids and liquids | 2 | 0 | 2 |
| | J69.0 | Pneumonitis due to food and vomit | 10 | 8 | 2 |
| | J85.1 | Abscess of lung with pneumonia | 2 | 1 | 1 |
| | J86 | Pyothorax | 2 | 2 | 0 |
| | J86.9 | Pyothorax with fistula | 3 | 1 | 2 |
| Other (n=72) | Other, all | | 72 | 45 | 27 |
| | A32.8 | Other forms of listeriosis | 3 | 2 | 1 |
| | A46 | Erysipelas | 5 | 4 | 1 |

| | | | | | |
|---|---|---|---|---|---|
| | A48.8 | Other specified bacterial diseases | 2 | 1 | 1 |
| | A49.0 | Staphylococcal infection, unspecified | 6 | 3 | 3 |
| | A49.9 | Bacterial infection, unspecified | 30 | 14 | 16 |
| | B96.2 | *Escherichia coli* [*E. coli*] as the cause of diseases classified to other chapters | 1 | 0 | 1 |
| | B99 | Other and unspecified infectious diseases | 2 | 2 | 0 |
| | G00.9 | Bacterial meningitis, unspecified | 1 | 1 | 0 |
| | J06.9 | Acute upper respiratory infection, unspecified | 2 | 2 | 0 |
| | L03.9 | Cellulitis, unspecified | 1 | 1 | 0 |
| | L97 | Ulcer of lower limb, not elsewhere classified | 2 | 1 | 1 |
| | M00 | Pyogenic arthritis | 1 | 1 | 0 |
| | M00.9 | Pyogenic arthritis, unspecified | 1 | 1 | 0 |
| | M46.3 | Infection of intervertebral disc (pyogenic) | 2 | 2 | 0 |
| | M86.1 | Other acute osteomyelitis | 1 | 0 | 1 |
| | M86.6 | Other chronic osteomyelitis | 1 | 1 | 0 |
| | M86.9 | Osteomyelitis, unspecified | 1 | 1 | 0 |
| | N49.8 | Inflammatory disorders of other specified male genital organs | 1 | 1 | 0 |
| | T81.4 | Infection following a procedure, not elsewhere classified | 8 | 7 | 1 |
| | T85.7 | Infection and inflammatory reaction due to other internal prosthetic devices, implants, and grafts | 1 | 0 | 1 |
| Peritonitis (n=73) | Peritonitis, all | | 73 | 50 | 23 |
| | K65 | Peritonitis | 7 | 7 | 0 |
| | K65.0 | Acute peritonitis | 52 | 36 | 16 |
| | K65.0*B95.6 | Acute peritonitis *Staphylococcus aureus* as the cause of diseases classified to other chapters | 1 | 0 | 1 |
| | K65.0*B96.2 | Acute peritonitis *Escherichia coli* [*E. coli*] as the cause of diseases classified to other chapters | 1 | 0 | 1 |
| | K65.8 | Other peritonitis | 8 | 6 | 2 |
| | K65.9 | Peritonitis, unspecified | 4 | 1 | 3 |
| Cardiac infection (n=27) | Cardiac infection, all | | 27 | 14 | 13 |
| | I33.0 | Acute and subacute infective endocarditis | 18 | 8 | 10 |
| | I33.0*B96.8 | Acute and subacute infective endocarditis | 1 | 0 | 1 |
| | I33.9 | Acute endocarditis, unspecified | 4 | 3 | 1 |
| | I38 | Acute endocarditis, unspecified | 2 | 1 | 1 |
| | I39.8 | Endocarditis, valve unspecified, in diseases classified elsewhere | 1 | 1 | 0 |
| | I40.0 | Infective myocarditis | 1 | 1 | 0 |
| GE infection (n=29) | Gastrointestinal infection, all | | 29 | 17 | 12 |
| | A04.7 | Enterocolitis due to Clostridium difficile | 11 | 7 | 4 |
| | A04.9 | Bacterial intestinal infection, unspecified | 1 | 0 | 1 |
| | A09 | Diarrhoea and gastroenteritis of presumed infectious origin | 1 | 1 | 0 |
| | K52.80 | Colitis [haemorrhagica] ex usu antibioticorum | 1 | 1 | 0 |

| | | | | | |
|---|---|---|---|---|---|
| | K61.1 | Rectal abscess | 1 | 0 | 1 |
| | K75.0 | Abscess of liver | 2 | 1 | 1 |
| | K80.0 | Calculus of gallbladder with acute cholecystitis | 5 | 3 | 2 |
| | K80.4 | Calculus of bile duct with cholecystitis | 1 | 0 | 1 |
| | K81.0 | Acute cholecystitis | 4 | 3 | 1 |
| | K83.0 | Cholangitis | 2 | 1 | 1 |
| Fungal infection (n=11) | Fungal infection, all | | 11 | 7 | 4 |
| | B37.7 | Candidal septicaemia | 7 | 4 | 3 |
| | B37.8 | Candidiasis of other sites | 2 | 2 | 0 |
| | B59 | Pneumocystosis | 1 | 1 | 0 |
| | J17.2 | Pneumonia in mycoses | 1 | 0 | 1 |
| Viral infection (n=7) | Viral infection, all | | 7 | 1 | 6 |
| | J10.0 | Influenza with pneumonia, influenza virus identified | 3 | 0 | 3 |
| | J10.1 | Influenza with other respiratory manifestations, other influenza virus identified | 2 | 0 | 2 |
| | J12.9 | Viral pneumonia, unspecified | 1 | 0 | 1 |
| | B25.0 | Cytomegaloviral pneumonitis | 1 | 1 | 0 |
| Tuberculosis (n=4) | Tuberculosis, all | | 4 | 1 | 3 |
| | A15.1 | Tuberculosis pulmonum solum cultura confirmata | 1 | 0 | 1 |
| | A15.6 | Tuberculous pleurisy, confirmed bacteriologically and histologically | 1 | 0 | 1 |
| | A18.0 | Tuberculosis of bones and joints | 1 | 1 | 0 |
| | A31.8 | Other mycobacterial infections | 1 | 0 | 1 |

**Table S3.** Adjusted Cox Regression Analysis for Infection-Related Mortality in Patients Treated With Maintenance Dialysis

| | Adjusted [a] HR (95% CI) | Adjusted [b] HR (95% CI) |
|---|---|---|
| Age (per 1-year increment) | 1.04 (1.03–1.05) | 1.04 (1.03–1.05) |
| Sex | | |
| Men | 1 | 1 |
| Women | 1.04 (0.91–1.20) | 1.01 (0.87–1.17) |
| Era | | |
| 2000–2004 | 1 | 1 |
| 2005–2009 | 0.81 (0.69–0.96) | 0.82 (0.69–0.97) |
| 2010–2014 | 0.68 (0.57–0.82) | 0.68 (0.57–0.82) |
| 2015–2019 | 0.49 (0.39–0.62) | 0.49 (0.39–0.62) |
| Cause of kidney disease | | |
| Glomerulonephritis | 1 | 1 |
| Polycystic kidney disease | 0.49 (0.30–0.81) | 0.62 (0.38–1.03) |
| Diabetes | 1.86 (1.43–2.43) | 1.66 (1.27–2.19) |
| Other | 1.76 (1.36–2.28) | 1.67 (1.29–2.17) |
| First treatment modality | | |
| Peritoneal dialysis | 1 | 1 |
| Hemodialysis | 0.99 (0.84–1.16) | 0.77 (0.64-0.92) |
| Treatment modality at 90 days from start of dialysis | | |
| Peritoneal dialysis | 1 | 1 |
| Hemodialysis | 0.89 (0.76–1.04) | 0.74 (0.63-0.88) |
| Weight status BMI (kg/m$^2$) | | |
| <25 normal | 1 | 1 |
| 25–29.9 overweight | 0.79 (0.68–0.93) | 0.81 (0.69–0.95) |
| >30 obese | 0.86 (0.73–1.01) | 0.83 (0.70–0.99) |
| Peripheral vascular disease | 1.68 (1.44–1.97) | 1.38 (1.16–1.63) |
| Coronary artery disease | 1.22 (1.05–1.41) | 0.93 (0.80–1.09) |
| Chronic heart failure | 1.89 (1.59–2.25) | 1.53 (1.27–1.85) |
| Cerebrovascular disease | 1.27 (1.05–1.53) | 1.16 (0.95–1.40) |
| Albumin (g/dL) | 0.63 (0.57–0.69) | 0.67 (0.60–0.75) |
| Creatinine (g/dL) | 0.94 (0.91–0.97) | 0.96 (0.93–0.98) |
| C-reactive protein (10g/L) | 1.03 (1.02–1.04) | 1.01 (0.99–1.02) |
| Hemoglobin g/dL | 0.95 (0.90–0.99) | 0.98 (0.93–1.03) |

a Adjusted for age and sex.

b Adjusted for age, sex, era of dialysis initiation, cause of kidney disease, weight status (BMI), peripheral vascular disease, coronary heart disease, chronic heart failure, cerebrovascular disease, serum albumin, creatinine, C-reactive protein, hemoglobin.

**Table S4.** Adjusted Cox Regression Analysis for Sepsis Mortality in Patients Treated With Maintenance Dialysis

| | Adjusted [a] HR (95%CI) | Adjusted [b] HR (95% CI) |
|---|---|---|
| Age (per 1–year increment) | 1.03 (1.02–1.04) | 1.03 (1.02–1.04) |
| Sex | | |
| Men | 1 | 1 |
| Women | 1.15 (0.93-1.44) | 1.12 (0.89-1.42) |
| Era | | |
| 2000-2004 | 1 | 1 |
| 2005-2009 | 0.76 (0.58-1.00) | 0.76 (0.58-1.01) |
| 2010.2014 | 0.76 (0.57-1.00) | 0.74 (0.55-0.98) |
| 2015-2019 | 0.51 (0.35-0.73) | 0.50 (0.35-0.71) |
| Cause of kidney disease | | |
| Glomerulonephritis | 1 | 1 |
| Polycystic kidney disease | 0.45 (0.19-1.06) | 0.61 (0.26-1.43) |
| Diabetes | 2.30 (1.48-3.58) | 1.93 (1.23-3.03) |
| Other | 1.85 (1.19-2.87) | 1.75 (1.12-2.71) |
| First treatment modality | | |
| Peritoneal dialysis | 1 | 1 |
| Hemodialysis | 1.39 (1.03-1.86) | 1.03 (0.76-1.41) |
| Treatment modality at 90 days | | |
| Peritoneal dialysis | 1 | 1 |
| Hemodialysis | 1.27 (0.95-1.71) | 1.02 (0.59-1.79) |
| Weight status BMI (kg/m$^2$) | | |
| <25 normal | 1 | 1 |
| 25-29.9 overweight | 0.87 (0.67-1.12) | 0.87 (0.67-1.13) |
| ≥30 obese | 1.03 (0.79-1.34) | 0.94 (0.71-1.24) |
| Peripheral vascular disease | 1.80 (1.40-2.32) | 1.36 (1.04-1.77) |
| Coronary artery disease | 1.11 (0.88-1.41) | 0.78 (0.60-1.01) |
| Chronic heart failure | 2.18 (1.66-2.86) | 1.63 (1.23-2.16) |
| Cerebrovascular disease | 1.35 (1.00-1.81) | 1.19 (0.88-1.60) |
| Albumin g/dL | 0.56 (0.48–0.65) | 0.62 (0.52-0.74) |
| Creatinine g/dL | 0.93 (0.89–0.97) | 0.95 (0.91–0.99) |
| C-reactive protein (10g/L) | 1.04 (1.02–1.06) | 1.02 (0.99-1.04) |
| Hemoglobin g/dL | 0.91 (0.85–0.98) | 0.96 (0.89-1.04) |

[a]Adjusted for patient age and sex.

[b]Adjusted for patient age and sex, era of dialysis initiation, cause of kidney disease, first treatment modality, peripheral vascular disease, chronic heart failure, cerebrovascular disease, serum albumin, creatinine, C-reactive protein, hemoglobin.

**Table S5.** Cause of Death by Main Categories for the 4 Study Periods From 2000 to 2019 as Fraction of all Patients Treated With Maintenance Dialysis

| | All<br>N=9,671<br>n=3,692 | 2000–2004<br>N=2,398<br>n=1,156 | 2005–2009<br>N=2,376<br>n=1,058 | 2010–2014<br>N=2,273<br>n=946 | 2015–2019<br>N=2,624<br>n=532 | *P* value |
|---|---|---|---|---|---|---|
| Infection-related death | 866 (9.0%) | 275 (11.5%) | 267 (11.2%) | 220 (9.7%) | 104 (4.0%) | <0.001 |
| Cardiovascular death | 1553 (16.1%) | 533 (22.2%) | 442 (18.6%) | 389 (17.1%) | 189 (7.2%) | <0.001 |
| Malignancy-related death | 338 (3.5%) | 75 (3.1%) | 91 (3.8%) | 103 (4.5%) | 69 (2.6%) | <0.001 |
| Death from other causes | 935 (9.7%) | 273 (11.4%) | 258 (10.9%) | 234 (10.3%) | 170 (6.5%) | <0.001 |

N, number of patients (%); n, number of deaths. The *P* values stand for chi-square test for trend

**Table S6.** Causes of Infectious Deaths over Four 5-Year Study Periods From 2000 to 2019 as Fraction of all Patients Treated With Maintenance Dialysis

| | All N=9,671 n=3,692 | 2000–2004 N=2,398 n=1,156 | 2005–2009 N=2,376 n=1,058 | 2010–2014 N=2,273 n=946 | 2015–2019 N=2,624 n=532 | *P* value |
|---|---|---|---|---|---|---|
| Sepsis | 333 (3.4%) | 106 (4.4%) | 94 (4.0%) | 91 (4.0%) | 42 (1.6%) | <0.001 |
| Pulmonary infection | 320 (3.3%) | 108 (4.5%) | 99 (4.2%) | 71 (3.1%) | 42 (1.6%) | <0.001 |
| Peritonitis | 73 (0.75%) | 24 (1.0%) | 26 (1.1%) | 15 (0.66%) | 8 (0.30%) | 0.001 |
| Other infectious deaths | 140 (1.4%) | 37 (1.5%) | 48 (2.0%) | 43 (1.9%) | 12 (0.46%) | <0.001 |

N, number of patients (%); n, number of deaths. The *P* values stand for chi-square test for trend

**Table S7**. Linear Regression Analysis of Time Trends for Infection-Related Mortality Based on Cox Hazard Ratios and Incidence Rates with 5-Year Restricted and Non-Restricted Follow-up From 2000 to 2019

| | Incidence rate, follow-up not restricted | Incidence rate, follow-up restricted to 5 years | Cox hazard ratio |
|---|---|---|---|
| Best-fit values | | | |
| Slope | -9,400 | -7,800 | -0,1630 |
| Y-intercept | 62,50 | 56,00 | 1,160 |
| X-intercept | 6,649 | 7,179 | 7,117 |
| 1/slope | -0,1064 | -0,1282 | -6,135 |
| Std. Error | | | |
| Slope | 1,105 | 1,039 | 0,007937 |
| Y-intercept | 3,025 | 2,846 | 0,02174 |
| 95% Confidence Intervals | | | |
| Slope | -14,15 to -4,648 | -12,27 to -3,329 | -0,1972 to -0,1288 |
| Y-intercept | 49,48 to 75,52 | 43,75 to 68,25 | 1,066 to 1,254 |
| X-intercept | 5,181 to 10,97 | 5,399 to 13,54 | 6,289 to 8,368 |
| Goodness of Fit | | | |
| R squared | 0,9731 | 0,9657 | 0,9953 |
| Sy.x | 2,470 | 2,324 | 0,01775 |
| Is slope significantly non-zero? | | | |
| F | 72,43 | 56,33 | 421,7 |
| DFn, DFd | 1, 2 | 1, 2 | 1, 2 |
| P value | 0,0135 | 0,0173 | 0,0024 |
| Deviation from zero? | Significant | Significant | Significant |
| Runs test | | | |
| Points above line | 2 | 2 | 2 |
| Points below line | 2 | 2 | 2 |
| Number of runs | 3 | 3 | 4 |
| P value (runs test) | 0,6667 | 0,6667 | >0,9999 |
| Deviation from linearity | Not Significant | Not Significant | Not Significant |
| Equation | Y = -9,400*X + 62,50 | Y = -7,800*X + 56,00 | Y = -0,1630*X + 1,160 |
| Data | | | |
| Number of X values | 4 | 4 | 4 |
| Maximum number of Y replicates | 1 | 1 | 1 |
| Total number of values | 4 | 4 | 4 |
| Number of missing values | 0 | 0 | 0 |

**Table S8.** Adjusted Cox Regression Analysis for Early Infection-Related Mortality Within one Year From Dialysis Initiation

| | Adjusted[a] HR (95% CI) | Adjusted[b] HR (95% CI) |
|---|---|---|
| Age (per 1-year increment) | 1.06 (1.04–1.07) | 1.05 (1.04–1.06) |
| Sex | | |
| Men | 1 | 1 |
| Women | 1.16 (0.89–1.52) | 1.22 (0.93–1.59) |
| Era | | |
| 2000–2004 | 1 | 1 |
| 2005–2009 | 0.73 (0.52–1.03) | 0.75 (0.53–1.06) |
| 2010–2014 | 0.74 (0.53–1.05) | 0.69 (0.49–0.98) |
| 2015–2019 | 0.49 (0.34–0.72) | 0.47 (0.32–0.69) |
| Cause of kidney disease | | |
| Glomerulonephritis | 1 | 1 |
| Polycystic kidney disease | 0.30 (0.10–0.89) | 0.49 (0.17–1.46) |
| Diabetes | 1.34 (0.80–2.23) | 1.12 (0.66–1.90) |
| Other | 1.85 (1.14–3.00) | 1.61 (0.99–2.62) |
| First treatment modality | | |
| Peritoneal dialysis | 1 | |
| Hemodialysis | 1.80 (1.23–2.65) | 1.05 (0.70–1.57) |
| Treatment modality at 90 days from start of dialysis | | |
| Peritoneal dialysis | 1 | 1 |
| Hemodialysis | 1.72 (1.14–2.59) | 1.19 (0.78–1.82) |
| Weight status BMI (kg/m$^2$) | | |
| <25 normal | 1 | 1 |
| 25–29.9 overweight | 0.69 (0.51–0.94) | 0.73 (0.53–0.99) |
| >30 obese | 0.80 (0.58–1.10) | 0.81 (0.58–1.14) |
| Peripheral vascular disease | 1.92 (1.43–2.56) | 1.59 (1.17–2.15) |
| Coronary artery disease | 1.06 (0.80–1.41) | |
| Chronic heart failure | 2.19 (1.62–2.97) | 1.86 (1.36–2.54) |
| Cerebrovascular disease | 0.93 (0.62–1.37) | |
| Albumin (g/dl) | 0.41 (0.34–0.48) | 0.50 (0.41–0.62) |
| Creatinine (g/dl) | 0.97 (0.92–1.02) | |
| C-reactive protein (10 g/L) | 1.06 (1.04–1.08) | 1.03 (1.01–1.05) |

| | | |
|---|---|---|
| Hemoglobin g/dl | 0.80 (0.73–0.87) | 0.89 (0.81–0.97) |

[a]Adjusted for patient age and sex.

[b] Adjusted for age, sex, era of dialysis initiation, cause of kidney disease, first treatment modality, weight status (BMI), peripheral vascular disease, chronic heart failure, serum albumin, C-reactive protein, hemoglobin

**Table S9** Time Trends of Infection-Related Risk of Death by Fine-Gray Model Taking into Account Kidney Transplantation and Death From Other Causes as Competing Risks

| Fine-Gray | 2000–2004 | 2005–2009 | *P* | 2010–2014 | *P* | 2015–2019 | *P* |
|---|---|---|---|---|---|---|---|
| **Without restricting follow-up time, accounting for other causes of death as competing risk** | | | | | | | |
| Unadjusted | 1 | 0.90 (0.78-1.04) | 0.14 | 0.72 (0.62-0.85) | <0.001 | 0.46 (0.37-0.57) | <0.001 |
| Adjusted for age and sex | 1 | 0.87 (0.76-1.01) | 0.06 | 0.69 (0.59-0.80) | <0.001 | 0.43 (0.35-0.53) | <0.001 |
| With multivariable adjustment [a] | 1 | 0.87 (0.75–1.00) | 0.056 | 0.68 (0.58–0.80) | <0.001 | 0.43 (0.34–0.53) | <0.001 |
| **Without restricting follow-up time, accounting for other causes of death and kidney transplantation as competing risk** | | | | | | | |
| Unadjusted | 1 | 0.96 (0.83–1.11) | 0.55 | 0.73 (0.62–0.85) | <0.001 | 0.39 (0.31–0.48) | <0.001 |
| Adjusted for age and sex | 1 | 0.89 (0.78–1.03) | 0.13 | 0.66 (0.57–0.78) | <0.001 | 0.36 (0.29–0.45) | <0.001 |
| With multivariable adjustment | 1 | 0.89 (0.77–1.03) | 0.12 | 0.66 (0.57–0.78) | <0.001 | 0.36 (0.29–0.45) | <0.001 |
| **Restricting follow-up time to 5 years, accounting for other causes of death as competing risk** | | | | | | | |
| Unadjusted | 1 | 0.92 (0.78–1.08) | 0.31 | 0.81 (0.68–0.96) | 0.018 | 0.53 (0.42–0.67) | <0.001 |
| Adjusted for age and sex | 1 | 0.88 (0.74–1.04) | 0.13 | 0.76 (0.63–0.91) | 0.002 | 0.49 (0.39–0.62) | <0.001 |
| With multivariable adjustment [a] | 1 | 0.88 (0.74–1.04) | 0.14 | 0.76 (0.64–0.91) | 0.002 | 0.50 (0.40–0.62) | <0.001 |
| **Restricting follow-up time to 5 years, accounting for other causes of death and kidney transplantation as competing risk** | | | | | | | |
| Unadjusted | 1 | 0.97 (0.82–1.15) | 0.71 | 0.83 (0.70–0.99) | 0.04 | 0.49 (0.39–0.61) | <0.001 |
| Adjusted for age and sex | 1 | 0.90 (0.76–1.06) | 0.21 | 0.75 (0.63–0.90) | 0.002 | 0.45 (0.36–0.56) | <0.001 |
| With multivariable adjustment [a] | 1 | 0.90 (0.76–1.07) | 0.22 | 0.75 (0.63–0.90) | 0.002 | 0.45 (0.36–0.57) | <0.001 |

[a] Adjusted for age at dialysis initiation, sex, cause of kidney disease, weight status (BMI), peripheral vascular disease, coronary artery disease, chronic heart failure, cerebrovascular disease, and serum albumin.
